# Supplementary material for: Knowledge, Attitude and Practices of Self-Medication Including Antibiotics among Health Care Professionals during the COVID-19 Pandemic in Pakistan: Findings and Implications
Source: Antibiotics (Basel). 2023 Feb 28;12(3):481. doi: 10.3390/antibiotics12030481 (PMC10044347; doi:10.3390/antibiotics12030481)
Supplement: Supplementary file 1 [file antibiotics-12-00481-s001.zip › antibiotics-2247353-supplementary.pdf]

Supplementary Table S1 – Study Questionnaire - Knowledge, attitude and practices of Self-medication among health care professional during COVID-19 pandemic in Punjab Pakistan

Section 1 **Demographic details of study participants**

Name of Institution …………………………………….
Name of Distirct…………………………………………

**Q1**	**Gender**
1	Male
2	Female
**Q2**	**Age (Years)**
1	20-30
2	31–40
3	41–50
4	51–60
**Q3**	**Residence**
1	Urban
2	Rural
**Q4**	**Marital status**
1	Single/divorced/widow
2	Married
**Q5**	**Occupation**
1	Doctors
2	Pharmacist
3	Nurse
4	Laboratory personnel
5	Physiotherapist
6	Health assistants/technicians
7	Others
**Q6**	**Education level?**
6	Diploma
7	Bachelor's degree
8	Post graduation/specialization
**Q7**	**Working department**
1	COVID-19 ward
2	Emergency
3	Medicine
4	Surgery
5	Pediatrics
6	Gynae
7	Cardiac
8	Others
**Q8**	**Hospital Type**
1	Tertiary care
2	Secondary care
3	Primary care
**Q9**	**Working experience**
1	1-3 years
2	4-7 years
3	8-12 years
4	More than 12 years

<u>Section 2 Knowledge of self-medications among health care professionals</u>

(Please select one option from Yes, No and Don't Know)

Questions	Yes	No	Don't know
Self-medication is defined as self-consuming of medications without getting advice from a physician			
Self-medication is safe?			
All medications (PRESCRIPTION OTC, HERBAL) have adverse effects?			
Increasing or decreasing medications dose without a doctor consultation can be dangerous?			
In case of adverse effects, physician help must be sought			
Using medications with unknown substances in patient with liver and kidney disease is dangerous			
Self-medication can mask signs and symptoms of some disease?			

<u>Section 3 Attitude of health care professionals about self-medications</u>

(Please select one option from strongly agree, Agree, Uncertain, Disagree, Strongly Disagree)

Questions	Strongly agree	Agree	Uncertain	Disagree	Strongly disagree
Self-medication is part of self-care					
No need for training to use self-medication					
Health care professionals are able to diagnosing different diseases					
Health care professionals are able to treat different diseases					
Do you recommend self-medications to others					
Easy access to healthcare information and facilities are the main causes that health care professionals use self-medications					
The availability of OTC medicines and belief on its safety leading me to use self-medications					

<u>Section 4 Practices of self-medications among health care professionals</u>

(Please select one option from below)

Self-medication during COVID-19?

1 Yes

2 No

Reason for self-medication?

1 No symptoms

2 Cold or flu

3 COVID-19 preventives

4 COVID-19 symptoms

5 COVID-19 positive

6 Consume it regularly

7 Other reason

Symptom improvement

1 All symptoms improved
2 Many symptoms improved
3 Some symptoms improved
4 One symptom improved
5 No improvement

<u>Section 5</u> <u>Commonly use drugs under self-medication</u>

Please select medicine(s) used under self-medication in last six months

**Class**	**Agents**	**Yes**	**No**
Antibiotics	1 Amoxicillin<br>2 Amoxicillin+clavulonic acid<br>3 Azithromycin<br>4 Ciprofloxacin<br>5 Levofloxacin<br>6 Moxifloxacin<br>7 Cefixime<br>8 Erythromycin<br>9 Doxycycline		
Antiallergic	1 Ibastine<br>2 Fexofenadine<br>3 Citrizine		
Leukotrienes receptor antagonist	Montelukast		
Ivermectin	Ivermectin		
Multivitamins	Multivitamins		
Zinc supplement	Zinc supplement		
Vitamin C + Calcium	Vitamin C + Calcium		
Antipyretics	Paracetamol		
Cough preparations	Cough preparations		
Herbal medicines	Herbal medicines		
Any others	Others		

.
